# Mesenchymal stem cells-derived exosomes: novel carriers for nanoparticle to combat cancer

**DOI:** 10.1186/s40001-023-01556-y

**Published:** 2023-12-09

**Authors:** Reza Abbasi, Raziye Momen Mesgin, Fereshteh Nazari-Khanamiri, Nima Abdyazdani, Zeynab Imani, Shabnam Pirnezhad Talatapeh, Aidin Nourmohammadi, Vahid Nejati, Jafar Rezaie

**Affiliations:** 1 Department of Biology, Urmia University, Urmia, Iran; 2 Solid Tumor Research Center, Cellular and Molecular Medicine Research Institute, Urmia University of Medical Sciences, Shafa St, Ershad Blvd, Urmia, Iran; 3 Stem Cell Research Center, Tabriz University of Medical Sciences, Tabriz, Iran; 4 Student Research Committee, Tabriz University of Medical Sciences, Tabriz, Iran

**Keywords:** Mesenchymal stem cells, Exosomes, Nanoparticles, Cancer therapy

## Abstract

**Background:**

The advancement in novel cancer therapeutics brought a platform combining the properties of exosomes with nanoparticles to precision medicine. The novel therapeutic approach aim is cancer-targeted therapy. Exosomes from mesenchymal stem cells (MSCs-Exo) exhibit unique properties in cancer therapies, which makes them an ideal tool for delivering therapeutic agents into tumor cells.

**The main body of the abstract:**

The key role of natural MSCs-Exo is controversial in cancer therapy; however, they can be engineered at their surface or cargo to serve as a smart drug delivery system for cancer-targeted therapy. In the last few years, researchers harnessed nanotechnology to enforce MSCs-Exo for cancer management including, tumor cell tracking, imaging, and tumor cell killing. Different nanoparticles such as gold nanoparticles have particularly been incorporated into MSCs-Exo, which showed an efficient accumulation at the site of tumor with improved anticancer impact. These findings indicate that a hybrid of exosomes–nanoparticles may serve as combination therapy for the effective removal of cancers.

**Short conclusion:**

Although exhibiting impressive potential, the use of nanoparticle-loaded MSCs-Exo as a drug-delivery tool has been troubled by some challenges, therefore, translation to clinic prerequisites further scrutiny. In this review, we focus on nanoparticle-loaded MSCs-Exo as a new cancer therapy and discuss engineered MSC-Exo for target therapy.

## Background

**Fig. 1:**
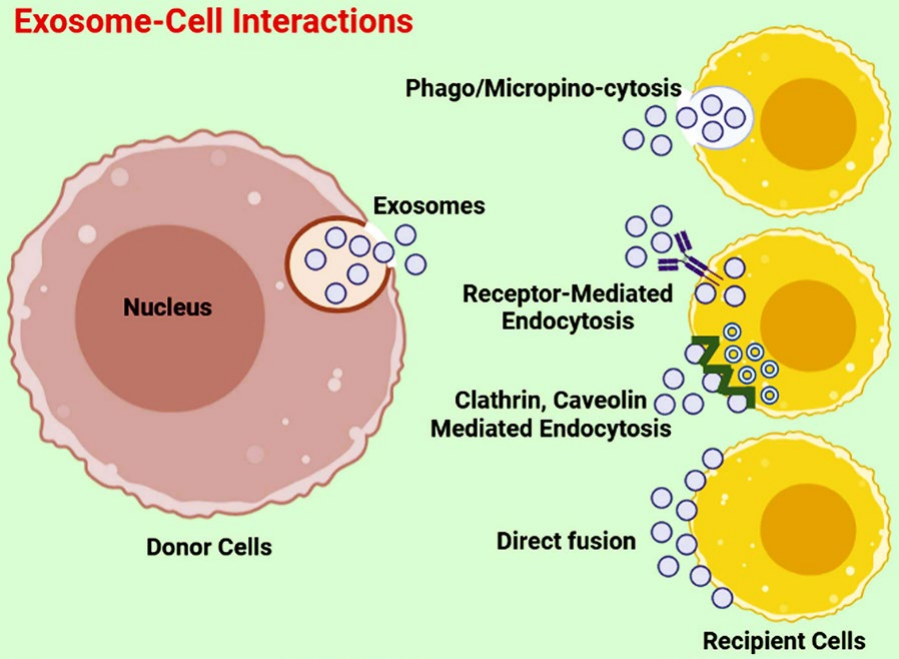
Interaction of exosomes with recipient cells. Once exosomes are secreted from cells, they can interact with recipient cells through internalization way (phagocytosis, micropinocytosis, and different endocytosis), receptor–ligand interaction, and direct fusion with the plasma membrane of recipient cells. Upon interaction with cells, exosomes can participate in cellular signaling and functions

Cancer is a significant global health challenge due to its devastating effects, including high rates of recurrence and mortality [1, 2]. In 2023, about 1.9 million incidences and 609,820 cancer-related mortality have been estimated in the United States [3]. To combat this, several methods including chemotherapy, radiation, and surgery are commonly being utilized [4, 5]. However, despite significant advances in medical technology in cancer therapy, cancer metastasis and recurrence remain the main challenge [6]. In recent years, stem cell therapy has emerged as a viable treatment option for various types of cancer. Mesenchymal stem cells (MSCs), adult stem cells, can self-renew and differentiate into several types of cell lines [7]. Originally found in the bone marrow [8], MSCs can be found in various body tissues and have the potential to contribute to tissue regeneration by differentiating into endodermal, ectodermal, and mesodermal cell lines [9]. In addition, these cells can regulate tumorigenesis via different signaling pathways; they may inhibit or promote tumors, which is controversial in the literature [10]. MSCs are capable of migrating towards damaged tissue and releasing bioactive substances, such as cytokines, growth factors, and extracellular vesicles (EVs), which offer a range of therapeutic benefits. These include immune suppression, anti-inflammatory properties, anti-apoptosis, antifibrosis, and angiogenesis [11]. MSCs can produce different EVs for instance exosomes to regulate different cellular processes from hemostasis to metastasis. Multiple studies in recent years have demonstrated that exosomes derived from MSCs (MSCs-Exo) may be involved in cancer development. However, there is ongoing debate surrounding the precise role of MSCs-Exo in cancer, as some studies have suggested that they could both promote and inhibit cancer progression [10]. The literature on exosome-based drug delivery systems shows a variety of cell sources are being used to load therapeutic agents onto their exosomes [12, 13]. However, this approach is in its infancy with faces challenges regarding selecting a suitable cell source and loading methods. Due to the useful advantages associated with MSCs-Exo, Scientists have been exploring the potential of using MSCs-Exo as a means of delivering therapeutic agents to tumor cells in both laboratory and animal studies in recent times [14, 15]. Previous research showed the successful creation of a therapy using a combination of MSCs-Exo and NP, demonstrating their potential in tissue repair [16]. In addition, Zhao et al. showed that combining exosome with engineering technology may increase drug targeting capacity of them to tumors [17]. On the other hand, various nanoparticles have been heavily investigated for cancer therapy [18, 19]. Nanoparticles could be used for tumor cells death or can deliver therapeutic agents to tumor cells [20, 21]. Recently, there has been a rising interest in merging nanoparticle therapy with exosome therapy to combat cancer metastasis. This review paper focuses on the latest information on the utilization of MSCs-Exo as a new drug delivery technique for treating tumors, particularly the utilization of nanoparticle-loaded MSCs-Exo. Additionally, the paper discusses the use of engineered MSCs-Exo for targeted therapy (Fig. 1).

## Exosomes loading methods

**Fig. 2:**
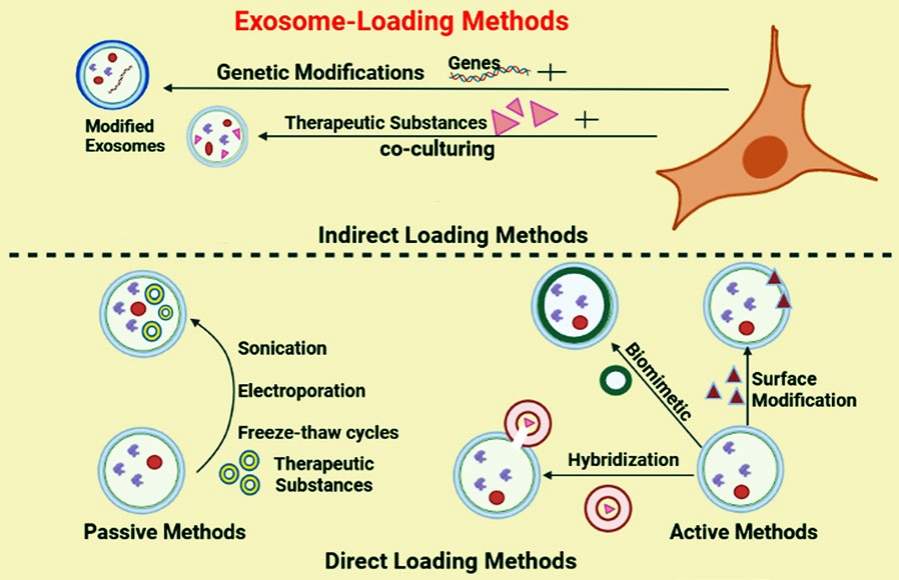
Different methods are used to load various therapeutic agents into exosomes. In general, exosomes are loaded with therapeutic agents by two methods, including indirect methods and direct methods. For example, in indirect methods, cells are genetically modified or co-cultured with therapeutic agents to produce exosomes containing an optional gene or therapeutic agent. In the direct method, exosomes are isolated from cell media and then therapeutic agents are inserted into them. Direct methods may divided into passive and active methods

Exosomes can be altered to serve drug delivery system for specific therapeutic aims [22]. Modifications of exosomes involves incorporating of therapeutic agents and other drugs into them, as well as altering the exosomal surface charge to facilitate rapid drug uptake [23]. Various loading techniques have been advanced to enhance the effectiveness of exosomes in cancer therapy [24, 25]. Typically, these strategies can be categorized as either indirect approaches that involve endocellular loading, or direct approaches that involve extracellular loading (Fig. 2). By endocellular loading methods, cargo is usually delivered into the exosome-producing cells. After being enveloped into exosomes, the exosomes containing cargo are collected for therapeutic application [26]. Methods for enhancing exosome therapeutic potential involve modifying parent cells to produce exosomes that overexpress certain biomolecules on the surface or inside the vesicles. Endocellular loading approaches typically involve co-incubation or genetic modification of parent cells to load genes or cargo into exosomes. Extracellular loading methods, on the other hand, involve directly loading cargo into exosomes isolated from cells using active techniques such as electroporation, sonication, incubation, freeze–thaw, or passive methods such as surface modification, hybridization, and biomimetic approaches (Fig. 2) (Table 1). Effective loading of therapeutic cargo into MSCs-Exos is an actual vital step. Now, investigators used various loading methods for several types of therapeutic cargo [27]. Transfection method is the most frequently used technique to load RNAs into exosomes. For example, in a study plasmids were used to transfect miR-122 into adipose-derived MSCs. Results showed that miR-122 molecules were effectively enriched in exosomes [28]. The electroporation method practices an electric field to form impermanent hydrophilic pores on the exosomes membrane to load the therapeutic cargo into them [29]. Gomari et al*.* used electroporation method to encompass doxorubicin into the MSC-Exo successfully. The loading effectiveness was calculated by a spectrophotometer [30]. However, some studies have pointed out that electroporation may induce RNA aggregation and fluctuations in the exosomes morphology [31]. Overexpression is frequently used for the loading of proteins in MSCs-Exo. Transfection attaches gene fragments to control cell protein synthesis. The target protein can be loaded into the MSCs-Exo through isolation and purification. This method is theoretically mature and easy to work. For example, Huang et al*.* effectively loaded pigment epithelium-derived factor (PEDF) into MSCs-Exo by overexpression and recognized the expression of PEDF in them via western blotting [32]. Modifying the targeting peptide on the surface of MSCs-Exo is an effective and direct method to expand the targeting capacity of MSCs-Exo [33]. In the study, researchers used the IMTP (ischemic myocardium-targeting peptide) motif CSTSMLKAC on MSC-Exo membrane to target the ischemic myocardium. The results of study revealed that IMTP exosomes considerably enhanced the targeting capability [34]. Among them, two of the simplest techniques for manipulating exosomes are incubation and freeze–thaw cycles. For example, Tian et al*.* employed a cyclo(Arg-Gly-Asp-D-Tyr-Lys) peptide [c(RGDyK)] to modify the surface of MSCs-Exo, enhancing their targeting capabilities in cerebral ischemia therapy [35]. Another study conducted by Kamerkar et al*.* focused on engineered EVs for the treatment of pancreatic cancer. The researchers employed electroporation to introduce siRNA or shRNA molecules that target KRASG12D, a common mutation in the KRAS GTPase that is associated with pancreatic ductal adenocarcinoma (PDAC)[36].

**Table 1:** Exosomes loading methods

Example		Advantage	Disadvantage
Direct methods			
Passive	Electroporation: creating an electric field in the microvesicles membrane to increase membrane permeability	Loading↑	Complications in changing cellular gene expression
		Safer↑	
	Extrusion: a combination of two membranes	Fast transfer ↑	Change the membrane proteins in EVs cytotoxicity↑
	Freeze–thaw: combination of drug with extracellular vesicle in liquid nitrogen at -80 temperature	Simple and loading↑	Loading ↓ increase the size of EVs and accumulation of exosomes
	Sonication: creating a mechanical cut using an ultrasound probe	Loading ↑	Damage the EVs structure
Active	Hybridization: Combining extracellular vesicles with nanovesicles	Loading↑	Potential toxicity
	EVs surface modifying: making connections between the ligand of the extracellular vesicle and the membrane of the target molecule	Loading↑	Potential toxicity↑
	Biomimetic EVs production: combination of metallic or inorganic nanoparticles with extracellular vesicles	Loading↑	Potential toxicity↑
Indirect methods			
Co-incubation: modifying parent cells with drugs and transferring and encapsulating them in cells		Loading↑	Aggregation therapeutic drugs↑
		Safer↑	
		Simple and cheap	
Gene editing (transfection): plasmid or vector transfer for protein production		Loading↑	Sensitivity and biocompatibility problem
		Safer↑	

Microfluidic technology has emerged as a pivotal loading method, significantly influencing the progression of biomedical research [37, 38] due to its advantages such as small size distribution, lower polydispersity index, heightened encapsulation and loading efficiencies, enhanced batch-to-batch uniformity, and facile scalability [39]. The current landscape of microfluidic techniques offers unparalleled opportunities for manipulating drug delivery [40] technologies like localized surface plasmon resonance (LSPR) and atomic force microscopy (AFM), a versatile scanning probe microscope, empower the visualization and characterization of the biomolecular composition of tumor-derived exosomes [40]. These methodologies exhibit the potential to sensitively detect exosomal surface proteins, providing fast, accurate, and reliable results with substantial diagnostic value [41]. In the pursuit of enhancing targeted delivery efficiency for exosomes in treating brain diseases, the optimization of exosomes through engineering technology proves to be an effective strategy. Notably, exosomes possess the ability to traverse the blood–brain barrier (BBB) [42, 43]. As an example by Kim et al., T7-exo stands out as an efficient carrier of AMO-21 into glioblastoma, showcasing its potential utility in glioblastoma therapy development [44]. Moreover, the genetic modification of autologous exosomes to carry ligands specific to receptors enhances cargo delivery stability [45]. Illustratively, in a system where Fe65-engineered exosomes from hippocampal neurons were utilized, the delivery of Cory-B to the brains of Alzheimer's disease (AD) mouse models was achieved. This targeted exosome-based delivery system holds promise as a compelling approach for AD treatment [43].

For convenience, the exosomes loading methods are given in Table 1.

## MSCs-Exo

There is evidence that MSCs can produce exosomes in large-scale [46]. In 2010, for the first time, MSCs-Exo were studied in the myocardial ischemia/reperfusion injury in an in vivo, then followed by many studies focusing on the function of these exosomes in several diseases [47]. Similar to other exosomes, MSCs-Exo have the same morphological features and separation and storing procedures [48]. Regarding exosomal markers, MSCs-Exo have common surface markers including CD9, CD63, and CD81; and also contain MSCs surface markers, like CD44, CD29, CD90 and CD73. In addition, these exosomes contain various biomolecules like exosomes from other cell sources [49]. MSCs-Exo have the unique characteristics that make them ideal for bio-application in the treatment of diverse human diseases (nano-carrier) such as low immunogenicity, biosafety, nano-size, long circulation half-life, ideal biocompatibility, outstanding penetration capability, and high uptake rate [49]. In addition, MSCs can be isolated from various tissues and grow in lab easily for the mass-production of exosomes [50]. Native MSCs-Exo can stimulate or prevent cancer cells, whereas engineered MSCs-Exo are involved in the destruction of cancer growth and development via the delivery of numerous therapeutics molecules containing miRNAs, specific siRNAs, drugs, anti-miRNAs, and proteins. In the next section, we focus of the therapeutic application of MSCs-Exo in delivering nanoparticles to cancer cells.

## Nanoparticle-loaded MSCs-Exo for targeted therapy

**Fig. 3:**
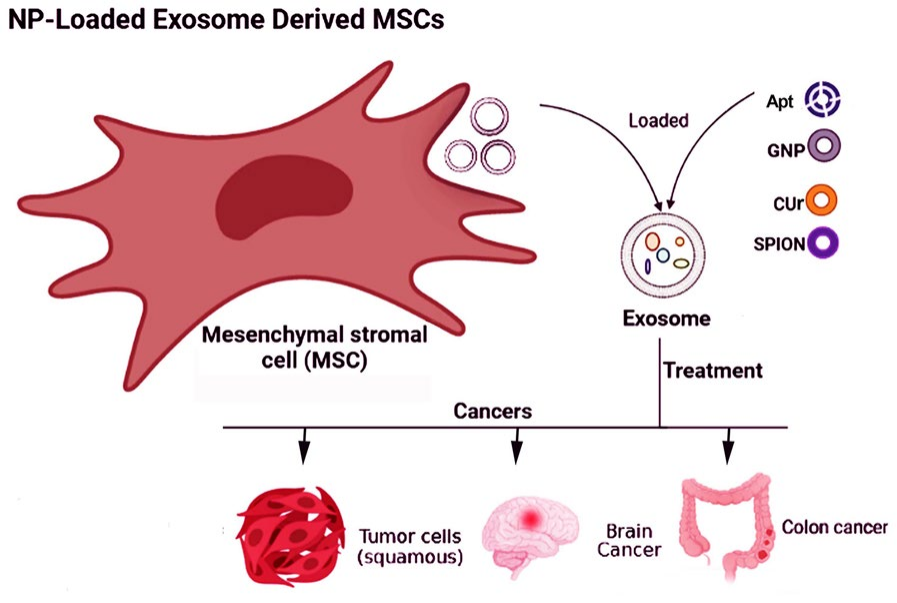
Application of nanoparticle-loaded MSCs-Exo for targeted therapy. Nanoparticles can be incorporated into MSCs-Exo to suppress and image tumor cells effectively. *Apt* aptamer, *Cur* curcumin, *GNP* gold nanoparticles, *NP* nanoparticles, *SPION* superparamagnetic iron oxide nanoparticles

Nanoparticles have demonstrated significant potential as carriers for delivering drugs owing to their small size, large surface area, and capability to attach targeting molecules [51]. By incorporating nanoparticles into exosomes obtained from MSCs, it is possible to specifically deliver them to targeted cells or tissues, making them an attractive approach for cancer-targeted therapy (Fig. 3) (Table 2). When these exosomes reach their target cells, they transmit information through binding to receptors or internalization [52]. Achieving clinical effectiveness with minimal amounts of nanoparticles while ensuring safety is an important objective. In a study, it was shown that gold nanoparticles (GNPs) coated with glucose were effectively taken up by MSCs-Exo through an active and energy-dependent mechanism. The team used a mouse model to track the labeled exosomes administered nasal way, and they observed that these exosomes showed a significant accumulation at the site of a brain injury within 24 h. This accumulation was higher compared to the random movement and clearance observed in control animals. The labeling method for exosomes has significant potential as a valuable diagnostic apparatus for various brain disorders and may improve treatments for neuronal regeneration [53]. More recently, a study also revealed that hybrid nanoparticles, which combined the features of exosomes and dendrimers, improved the uptake of dendrimers by cells without causing significant toxicity. By utilizing these hybrid nanoparticles, the researchers achieved enhanced delivery of oligonucleotides to cancer cells, surpassing the delivery efficiency of dendrimers alone by more than twofold. This research demonstrated the integration of exosomes of MCF-7 cells and dendrimers to produce a versatile nanoparticle platform, offering a new approach to nanoparticle design [54]. Using a mouse model of colon adenocarcinoma called C26, researchers observed that a single intravenous injection of doxorubicin (a chemotherapy drug) encapsulated in exosome–aptamer complexes significantly suppressed tumor growth when compared to free doxorubicin. The study used BALB/c mice and found that the exosome–aptamer complexes derived from MSCs loaded with doxorubicin (MSCs-Exo-Apt-Dox) showed improved efficacy in inhibiting tumor growth [55]. Nisim Perets et al*.* established a protocol to coat glucose onto GNPs to prepare MSC-Exo. In keeping, they utilized this method to monitor the movement and targeting behaviors of MSC-Exo following administration intranasally. These investigations covered various brain conditions such as stroke, autism, Parkinson's disease, and Alzheimer's disease, involving the utilization of exosomes loaded with nanoparticles [56]. Oded Cohen and colleagues investigated these particles in MSCs-Exo and A431 squamous cell carcinoma line-derived exosomes (A431-exo), both of which hold promise for cancer treatment. They used GNPs for labeling exosomes to target and track tumors and their distribution within the body. Findings indicate that MSCs-Exo, in particular, exhibits enhanced capabilities for tumor-targeted therapy [57]. In other research, researchers incorporated superparamagnetic iron oxide nanoparticles (SPIONs) and curcumin (Cur) into exosomes derived from MSCs. The modified exosomes were found to be capable of effectively passing through the blood–brain barrier and exhibited promising results in targeted imaging and glioma therapy. [58]. More recently, Yang et al. loaded cobalt sulfide quantum dots (CoS QDs) into MSCs-Exo to study anticancer effects. Results showed that MSCs-Exo loaded with CoS QDs could efficiently increase ROS in bladder cancer cells. In addition, in vitro and in vivo results showed that these exosomes exerted remarkable anticancer properties through chemodynamic and photothermal impacts [59]. Overall, these findings indicate that incorporating nanoparticles into MSCs-Exo has more anticancer effects. In addition, it seems that these exosomes are useful for targeted therapy and tumor cells imaging as well as tracking. To the best of our knowledge, there are few studies and further research on these exosomes is necessary to extend our knowledge of the usefulness of MSCs-Exo loaded nanoparticles in cancer-targeted and effective therapies.

**Table 2:** Studies related to nanoparticle-loaded MSCs-Exo for targeted therapy

Cell type	Nanoparticles	Disease	Loading method	Results	References
MSCs	Gold nanoparticles (GNPs)	Brain injury	Glucose enters cells through an energy-dependent process that is facilitated by a specific protein called GLUT-1	Significant accumulation of labeled exosomes at the site of brain injury within 24 h	[54]
MSCs	Gold nanoparticles (GNPs)	Various brain disorders	Glucose enters cells through an energy-dependent process that is facilitated by a specific protein called GLUT-1	Promising diagnostic tool for brain disorders- Potential for enhancing neuronal recovery	[57]
Human MSCs, A431 squamous cell line	GNPs	Cancer	Glucose enters cells through an energy-dependent process that is facilitated by a specific protein called GLUT-1	Enhanced capabilities of MSCs-Exo for tumor-targeted therapy	[58]
MSCs	Cobalt sulfide quantum dots (CoS QDs)	Bladder cancer	Electrotransfection	These exosomes specifically induced the increase of reactive oxygen species (ROS) concentration in bladder cancer cells	[59]
MSCs	Superparamagnetic iron oxide nanoparticles (SPIONs), curcumin	Glioma	Click chemistry	–Effective crossing of the blood–brain barrier- Positive outcomes in targeted imaging and glioma therapy	[59]

## Engineered MSC-Exo for target therapy

**Fig. 4:**
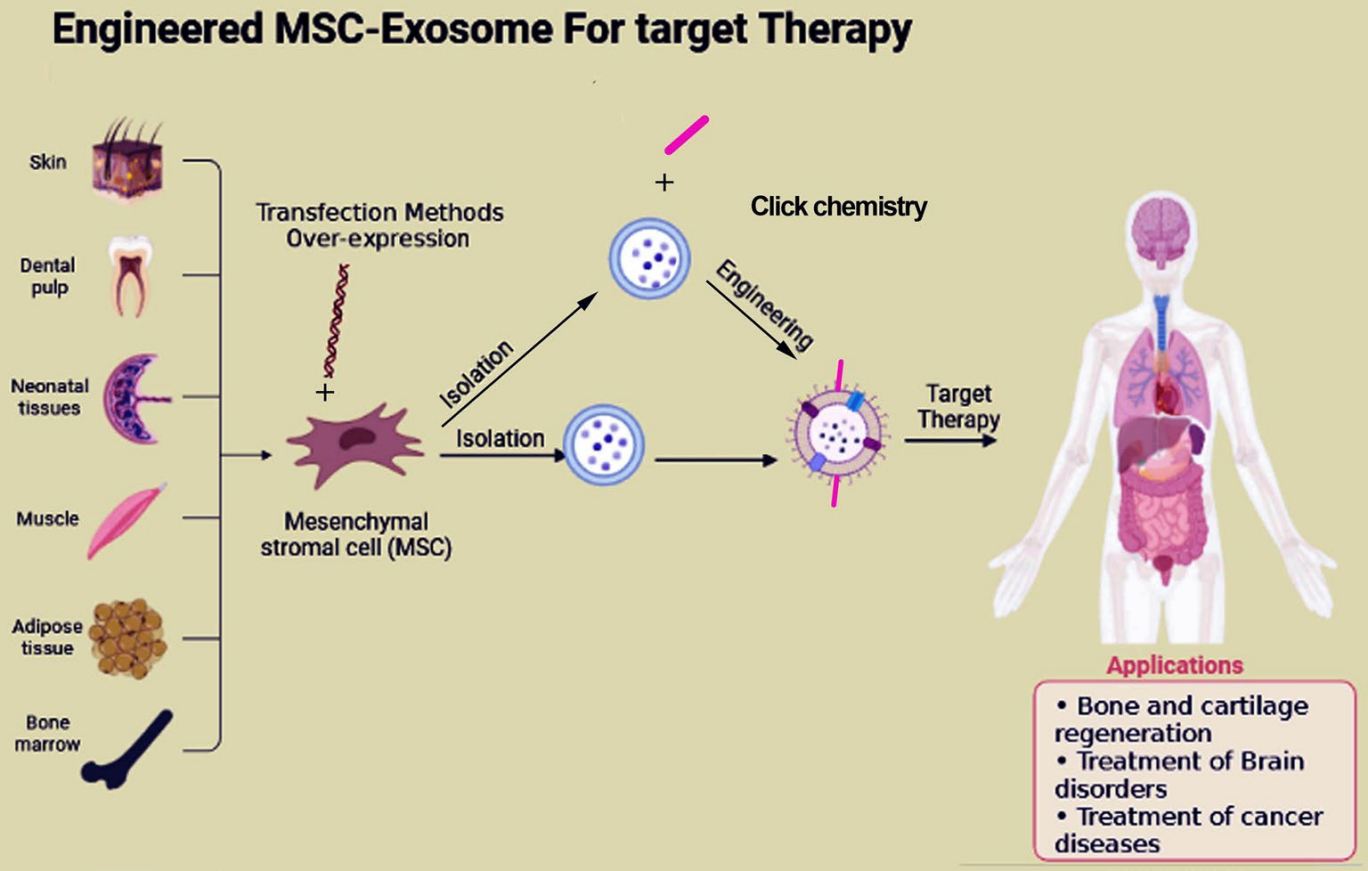
Engineered MSC-Exo for target therapy. Targeted therapy, an innovative approach to treating cancer, shows promise in combating tumors. In this regard, the surface of MSCs-Exo is genetically or chemically engineered to promote their targeted potential of them. For genetically modification, MSCs are transfected with target gene to produce optional MSCs-Exo, for chemically modification, isolated MSCs-Exo are modified. At the same time, this method is used for cancer imaging and precision therapy. These exosomes called engineered MSCs-Exo can target sites of damage or tumor when systematically administrated

Advancements in genetic engineering and click chemistry techniques have opened up exciting possibilities for tailoring MSC-Exo to deliver specific therapeutic payloads, leading to the emergence of engineered MSC-Exo target therapy as a promising avenue for precision medicine [60] (Fig. 4). Targeted therapy, an innovative approach to treating cancer, shows promise in combating tumors involving MSCs-Exo. The ideal drug carrier should specifically target cells or tissues while minimizing systemic side effects. Targeted therapies offer hope in overcoming tumor resistance and cancer metastasis, with minimal non-targeting effects. In this regard, in a study, researchers investigated the probability of advancing a vector based on MSCs-Exo for compacting oral squamous cell carcinoma (OSCC) to carry Cabazitaxel (CTX) /TRAIL combinations. They showed that genetically modified MSCs released exosomes that contain both TRAIL and CTX. MSCs-Exo/CTX showed an effective synergistic impact and a highly efficient pharmacological suppression on tumor cells, as confirmed by the following mouse model [61]. Scientists used bone marrow MSCs (BM-MSCs) derived exosomes to specifically target cancer to modify the tumor cell kinetics in pancreatic ductal adenocarcinoma (PDAC)[62]. BM-MSCs-Exo tagged with rabies viral glycoprotein (RVG) demonstrated enhanced targeting to the cortex and hippocampus following intravenous administration. Results present a novel approach to increase the delivery of exosomes for the treatment of Alzheimer's disease in an animal model [63]. In addition, by targeting exosomes to the cortex and hippocampus of mice, they observed significant improvements in learning and memory abilities, removed plaque deposition and Aβ levels, as well as normalized inflammatory cytokine levels associated with Alzheimer's disease [63]. The data suggest that modified BM-MSCs-Exo attenuates myocardial ischemia/reperfusion (I/R) injury in mice by delivering miR-182, which modulates the polarization status of macrophages during ischemia [64]. Through a transfection method, Wang et al. isolated BM-MSCs-Exo that overexpressed CTnI-targeted peptides on their surfaces. Engineered exosomes may be directed to the myocardial infarction (MI) zone by exploiting the concentration gradient of cardiac troponin I (CTnI), which is upregulated in this area. [65]. Jia and colleagues employed click chemistry to attach a neuropilin-1-targeted peptide (RGERPPR) onto the membrane of MSCs-Exo. This modification enabled efficient targeting of glioma cells [58]. In a different investigation, the IMTP motif CSTSMLKAC was incorporated into the surface of MSCs-Exo to target the MI zone. The results of the study revealed that IMTP exosomes showed a significant improvement in their targeting abilities [65]. To enhance the bone regeneration properties of MSCs, they were modified through the modification of BMP2 gene. This modification resulted in exosomes biogenesis with modified features and cargo (MSCs-BMP2-Exo). These exosomes demonstrated a significant ability to facilitate bone healing in a mouse model with bone defects [66]. Therefore, engineered MSCs-Exo showed promising results in the targeted therapy area.

## Clinical trials

Currently, ongoing clinical trials are studying the use of EVs/exosomes as carriers for therapeutic agents in various diseases. An analysis of the ClinicalTrials.gov database reveals the registration of a clinical trial in its Phase I stage. This clinical trial aims to determine the appropriate dosage and evaluate any potential adverse effects of iExosomes, which are MSCs-Exo containing KrasG12D siRNA, in the treatment of pancreatic cancer patients with metastasis to other areas of the body and characterized by the KrasG12D mutation (NCT03608631). Researchers have harnessed the immunomodulatory and regenerative properties of MSC-Exo to enhance the therapeutic outcomes of drug delivery. By modulating inflammation, promoting tissue repair, or influencing cell-signaling pathways, MSC-Exo can augment the efficacy of drug treatment in clinical trials.

## Conclusion and further perspective

Our work has led us to conclude that MSCs-Exo loaded with nanoparticles showed great promise for cancer therapy. They can be used for tumor cell tracking, imaging, and efficient killing. In addition, studies showed that these exosomes are easily captured with tumor cells, delivering nanoparticles into cellular cytoplasm. Administration of nanoparticles loaded MSCs-Exo may exert low systematic toxicity because nanoparticles are encapsulated by exosomes, further, protecting them from macrophage cleaning in the systematic system. Although there are limitations due to nanoparticles, this approach is in its infancy and few studies examined their efficacy. It is not clear which nanoparticle is suitable for loading into MSCs-Exo. Which method is universal and suitable for loading MSCs-Exo? Does nanoparticle interface with exosomes structure and function? What is the fate or/and interaction way of these exosomes with target cells? And what is a proper source for isolating MSCs-Exo? In addition, we have provided further evidence for the usefulness of engineered MSCs-Exo in targeted therapy, which showed that these exosomes can specifically target cells or tissues when systematically administrated. This feature makes them an ideal tool for precision imaging and treatment. Results so far have been very promising, however, for clinical applications, some challenges remain to be overwhelmed, which are discussed here as questions. Do engineered methods disrupt MSCs-Exo integrity? Do engineered methods modify MSCs-Exo cargo? What is the biodistribution of engineered MSCs-Exo? And which method is suitable for engineering MSCs-Exo? In addition, the advanced analytical techniques like Raman and FT-IR for the evaluation of exosome-based delivery systems have been reported. For example, Horgan et al. developed a metabolic labeling strategy utilizing deuterium for studying EVs through a confocal spontaneous Raman micro-spectroscopy system [67]. Raman scattering-based immunoaffinity, exploiting specific chemical fingerprints, and magnetic properties for exosome isolation and characterization have been explored. Previous studies have demonstrated the high sensitivity and specificity of the Raman method in detecting breast cancer in patients [68]. Fourier transform infrared (FTIR) spectroscopy is another method that has shown successful diagnosis in blood exosome samples from Alzheimer's patients [69].

## Data Availability

None.

## Abbreviations

AD: Alzheimer's disease
BALF: Bronchoalveolar lavage fluid
BM-MSCs: Bone marrow mesenchymal stem cell
CSF: Cerebrospinal fluid
CM: Conditioned medium
CTX: Cabazitaxel
Cur: Curcumin
CoS QDs: Cobalt sulphide quantum dots
EV: Extracellular vesicles
I/R: Ischemia/reperfusion
MSCs: Mesenchymal stem cells
MSCs-Exo: Exosomes from mesenchymal stem cells
MVBs: Multivesicular bodies
PDAC: Pancreatic ductal adenocarcinoma
PEDF: Pigment epithelium-derived factor
PTX: Paclitaxcel
RVG: Rabies viral glycoprotein
SCC: Squamous cell carcinoma
SPIONs: Superparamagnetic iron oxide nanoparticles
GNPs: Gold nanoparticles
LSPR: Localized SPR
AFM: Atomic force microscopy
BBB: Blood–brain barrier
DDS: Drug delivery system
FTIR: Fourier transform infrared
